# The influence of digital media on the success of a health care unit

**DOI:** 10.25122/jml-2018-0064

**Published:** 2018

**Authors:** Maria Radu, Gabriel Radu, Alexandru Condurache, Victor Lorin Purcărea

**Affiliations:** 1. Carol Davila University of Medicien and Pharmacy; 2. Universitatea de Medicina si Farmacie Carol Davila Facultatea de Medicina

**Keywords:** healthcare marketing, digital media, notoriety, social networking

## Abstract

Healthcare marketing extended in a surprising way, being indispensable to any healthcare unit that wishes to carry out its work by focusing on the needs and desires of patients. In the context of the medical activity taking more and more a commercial direction, the perception of the public about the profession of a physician should be considered, a perception which can be very easily negatively influenced by unethical marketing strategies. The notion of reputation refers to the perception of a group of people over a person or an entity, based on several criteria. These criteria have evolved over time with society and its needs. A company’s reputation plays a significant role because it is a key element in determining the success or failure of a company, regardless of the size of the business, be it a small company or a multinational. The first factor from which the building of a reputation starts is the relationship between the company and the client, namely the medical unit and the patient. Online reputation plays a determining role in establishing and maintaining a relationship with current or potential patients. Increasing the number of people who interact directly or indirectly with that company leads to increased brand visibility and an effective digital promotion. The Internet has made it easier for consumers to give a review or to comment on something. Thus, we can see a growing trend of people who rely on such reviews. An increasing number of patients choose to rely on existing assessments of doctors or clinics when it comes to making a decision regarding the choice of a medical service. In fact, more and more patients are looking for a doctor and choosing him “on the internet”. The Internet has opened the way for new opportunities in healthcare marketing. Social networks are frequented daily by millions of users worldwide, so digital media is the direction that all companies are taking when planning their marketing strategies.

In the present day, sanitary marketing has become very important, being indispensable to any healthcare unit that wishes to carry out its work by focusing on the needs and desires of patients.

In the context of the medical activity taking more and more a commercial direction, the perception of the public about the profession of a physician should be considered, a perception which can be very easily influenced negatively by unethical marketing strategies. Healthcare organizations have a duty to practice a responsible marketing communication [1].

For a long time, it has been considered that marketing is the tool that tries to sell people things they never knew they wanted. Many even consider ads to be manipulation tools. However, the real problems are represented by marketing communications that aim to raise fear or anxiety, that target vulnerable groups, the subliminal messages, which at first glance seem intelligent, yet being misleading, as well as the exaggeration or false allegations. All these aspects should be taken into account by marketing managers when they are doing their work because it contributes significantly to building a company’s reputation [2].

The notion of reputation refers to the perception of a group of people over a person or entity based on several criteria. These criteria have evolved over time with society and its needs.

A company’s reputation plays a significant role because it is a key element in determining the success or failure of that company, regardless of the size of the business, be it a small company or a multinational [3].

We live in the century of digitalization, and the Internet is the prime resource when it comes to information and communication. This made building a reputation easier and turned it into a much faster process. In the past, towards building a company’s reputation, the verbal recommendations, written publications, or TV and radio commercials contributed the most, and the process took place over a more extended period. At present, reputation is made much easier due to digitalization.

“Social networking” is a global phenomenon with millions of users worldwide. It is the way people keep in touch with each other very easily, by exchanging information, pictures, experiences, opinions. Social networks have thus become a valuable marketing tool.

The first factor from which the building of a reputation starts is the relationship between the company and the client, namely the medical unit and the patient. Online reputation plays a determining role in establishing and maintaining a relationship with current or potential patients. Increasing the number of people who interact directly or indirectly with that company leads to increased brand visibility and effective digital promotion. Besides, it is essential that the total number of positive reviews in the online environment exceeds that of negative comments. Building a good reputation online also contributes to an easy and transparent communication from the organization towards the patients. They want to communicate easily and get a quick response from the company.

Regarding the healthcare system, reputation plays a crucial role because it can influence the medical process and the patients’ behavior as well as their attitude towards the treatment or the physician. Patients build a set of expectations based on what they know about the healthcare unit, information received from other patients, or even based on the image built by the company and perceived favorably by them. Today, assessments of any service prior to purchasing it is important, and people will be even more interested proof of interest in something as important as health.

Regarding the private hospitals and clinics, is the one that positive or negative perception that patients will have when entering the health facility depends on the medical staff. Currently, because of the Internet, which is an inexhaustible source of information, the role of doctors has become more complicated. In addition to good professional training, they must have very good communication skills in order to relate well to patients. In the past, medical records and access to information that was of direct concern to them were reserved exclusively for healthcare professionals or those working in the healthcare field. Today, attitudes and thinking are entirely different, and it is necessary for both parties, patients and health professionals, to adapt to this change in order to improve their relationships and achieve the best results. Due to the above-mentioned concepts, those in which the doctor is the ultimate authority, the population segments that refuse to trust the medical community and the specialized personnel, as well as medical products or treatments has increased. It is one of the greatest challenges faced by the healthcare system nowadays. Healthcare staff must agree to become more involved in communicating with patients in both direct and online environments, as the reputation of the sanitary institutions depends on it.

The Internet has made it easier for consumers to give a review or to comment on something. Thus, we can see a growing trend of people who rely on such reviews. An increasing number of patients choose to rely on existing assessments of doctors or clinics when it comes to making a decision regarding the choice of medical services. In fact, more and more patients are looking for a doctor and choosing him “on the internet.” If until recently, the recommendation made by a satisfied patient was the best advertisement a doctor could hope for, today this concept must be understood at a completely different level since anyone can read the positive review of a satisfied patient, the information going beyond the barrier of each person’s close circle [4].

However, in this situation, the risk is represented by those patients who can never be satisfied or who refuse to accept the real possibilities of treatment and opt to post an unjustified negative opinion online, an opinion that can be read by anyone. In addition, people are different and consequently, patients have different criteria by which to appreciate a medical service because they have different expectations and desires. A service appreciated by a patient as being of poor quality can be considered by another mediocre, just as well as one considered mediocre by a particular consumer can be appreciated as being of good quality by another.

Sanitary institutions are adapting to the current trend and are giving great importance to communicating with patients through social networking. There is dedicated digital media staff who is responsible for creating content for posts, promoting the brand on the online platform, moderating discussions, evaluating reviews, or reviewing the success of online campaigns based on the number of views or hits of a certain post.

In Romania’s case, in the absence of a well-structured platform with a large number of physicians and clinics, the handiest options are using Google as a search engine and requesting opinions on the most frequented social networks.

Staff responsible for patient care must be kept up to date with their changing expectations and needs so that the healthcare unit can adapt their service offer so that the reputation of the institution will not be affected.

The Internet has opened the way towards new opportunities for healthcare marketing. Social networks are frequented daily by millions of users worldwide, so digital media is the direction that all companies are taking when planning their marketing strategies. Posts with attractive content, which keep in touch with the public, as well as positive patient reviews, are the key to the success of a healthcare unit regarding its presence online [5].

The health sector is undoubtedly one of the most important players in the social market, being not only the decisive factor in the general health of the population but also a major objective in research and development and a significant employer.

That is why health innovation is given a great deal of attention, with new health technologies contributing to a superior care quality for patients, and implicitly to the efficient and cost-effective development of health systems.

Continuous and constant information of the population on health risks has become an objective necessity and the Internet is today one of the most important sources of information in the field of health.

The current explosion of information technologies has become a decision-making factor in setting new global hierarchies, information, and communication going beyond the metaphor stage in the social fabric.

The development of medical technology and discoveries in medical science have led to a reshaping of the health sector through the development of medical informatics apps, designed to provide new and more effective solutions and through the creation of new directions for healthcare with profound implications for man and society.

The exponential growth of digital information has led to the emergence of more efficient, more transparent and faster healthcare services, closer to the needs of individuals and less expensive.

Due to the particularity and complexity of the medical field, web technologies and online marketing tools are mandatory conditions for achieving the objectives of modern medicine, and their use can become the decision maker in focusing the staff’s concerns and efforts towards patient needs, personalizing health services and improving the doctor-patient relationship.

Thanks to this explosion of information, patients tend to appreciate the quality of a health product or service not based on reality but based on their perceptions. Online healthcare consumers tend to be more educated but use online information in different ways and receive different responses from medical contact staff. Regardless of the way of responding, it is obvious that web technologies and online marketing tools tend to become more and more a constant and undisputed presence in health services. Proper implementation and use will be beneficial to both patients and the healthcare organization.

This is why the efforts made to explore patients’ behavior for interpersonal communication in electronic media in health services, towards technological and informational optimization of patient and healthcare communication processes, towards negative emotional modeling, patient-centered networks, and mostly towards investigating the influence of virtual communities on the reputation of healthcare organizations, are of some use.

Under these circumstances, the ability to anticipate accelerated changes in the healthcare system has become a strategic resource and healthcare marketing a modern solution for identifying the possibilities of innovation in providing healthcare services, starting from a thorough knowledge of the health market and from the proper use of new technologies, including those in the healthcare system generating interactive synergies.

Situated at the crossroad of marketing, economics and technology, online marketing is a modern approach to marketing in the context of the accelerated growth of the Internet resulting in an unlimited expansion – especially due to the many opportunities and benefits offered by the online environment compared to the traditional one, the online environment providing continuous and constant support to patients.

In healthcare, information and communication technologies make a decisive contribution towards improving competitiveness, increasing efficiency and reducing bureaucracy, thus contributing towards improving the health of the population overall. Also, we should not forget that access to education and healthcare is considered a fundamental right of the individual in all civilized countries.

## Conflict of Interest

The authors confirm that there are no conflicts of interest.
